# Effects of Animal-Assisted Therapy (AAT) in Alzheimer’s Disease: A Case Study

**DOI:** 10.3390/healthcare10030567

**Published:** 2022-03-18

**Authors:** Armando Gregorini, Angela Di Canio, Emanuele Palmucci, Marco Tomasetti, Marco B. L. Rocchi, Mariastella Colomba

**Affiliations:** 1 Department of Biomolecular Sciences, University of Urbino Carlo Bo, 61029 Urbino, Italy; marco.rocchi@uniurb.it (M.B.L.R.); mariastella.colomba@uniurb.it (M.C.); 2 Studio A.P., via 25 Aprile n. 8, 37030 Vago di Lavagno, Italy; angeladicanio@live.com; 3 Smallegade 24, 2.tv, 2000 Frederiksberg, Denmark; emanuele.palmucci@yahoo.it; 4 Department of Clinical and Molecular Sciences, Università Politecnica delle Marche, 60126 Ancona, Italy; m.tomasetti@univpm.it

**Keywords:** neurodegenerative disorders, cognitive and behavioral interventions, heart rate, systolic and diastolic blood pressure, MMSE, GDS

## Abstract

Alzheimer’s disease (AD) is a neurodegenerative disorder, characterized by cortical dementia and irreversibly progressive developments leading to a vegetative state and, finally, to death. Although many aspects of its etiology, diagnosis and treatment still remain obscure and the current approach to the disease mostly suffers from limited and low-efficiency therapeutic means, nevertheless, recent interventions have aimed at improving patients’ quality of life through nonpharmacological approaches, including animal-assisted therapy (AAT), arousing growing interest. In order to assess the physiological and neuropsychological effects of AAT on AD, 24 residents of a rest house in northern Italy were enrolled. The intervention consisted of one 45-minute AAT session per week over ten weeks. Twelve residents (six AD and six non-AD) received AAT and twelve (six AD and six non-AD) were controls. In order to evaluate the physiological and clinical effect of AAT on AD residents, three cardiac parameters, including the systolic and diastolic blood pressure and heart rate, were measured. Moreover, the neurocognitive and depressive states were assessed by the Mini Mental State Examination and the Geriatric Depression Scale, respectively. Analyses were performed by a four-way ANOVA model (including two ways for repeated measures) considering each main effect and interaction possible in the design. Our findings, despite the small sample size, suggest that AAT has a positive significant effect on physiological parameters and neurocognitive impairment, while no effect was observed on the depression level.

## 1. Introduction

“Dementia is one of the main causes of disability and dependence among older adults worldwide, constituting a public health priority due to its significant human and financial costs to society. Oxidative stress, mitochondrial damage, and synaptic damage are all implicated in dementia pathogenesis, playing an important role in the cognitive impairment and memory loss of older individuals with Alzheimer’s disease” [1].

Alzheimer–Perusini disease (from the names of the two scientists who first described it), commonly referred to as Alzheimer’s disease (AD), is a degenerative pathology of the nervous system that mainly involves the cerebral cortex by the formation of extracellular senile plaques and intraneuronal neurofibrillary tangles. AD is a very complex disorder (without a cure), whose causes and treatment are still under investigation [2,3,4,5,6,7,8,9,10], as a lack of understanding of the disease mechanism hinders the development of efficacious therapeutic approaches. Medical literature identifies two forms of AD: (i) late-onset Alzheimer’s disease (LOAD), affecting individuals after sixty-five years of age and being the most widespread form; (ii) early-onset familial Alzheimer’s disease (EOFAD), with early onset before 65 years, predominantly of genetic origin, but is less frequent than LOAD. 

The behavioral and psychological manifestations of AD are a source of suffering and discomfort for the patients and their family members, often causing the institutionalization of the affected persons.

Depressive syndrome is the most common psychotic disorder seen in individuals with Alzheimer’s disease. However, the association between depression and dementia has many possible correlations, resulting in a large overlap of several clinical frameworks, which may make it difficult to diagnose and understand the etiopathogenesis of depressive syndrome in patients with AD [11].

The psychological and rehabilitative approach to patients with dementia promotes (i) cognitive interventions [12] aimed at delaying the progressive loss of functions by maintaining the residual cognitive abilities of the subject and (ii) behavioral interventions aimed at improving the patient’s quality of life [13,14,15]. Among these, increasingly more frequently, nonpharmacological therapies are being recognized as a useful addition to traditional ones [16], although further research is still needed to continue to strengthen the evidence of their effectiveness. Nonpharmacological therapies include, among others, animal-assisted therapy (AAT), a goal oriented, planned and structured therapeutic intervention incorporating animals (dogs or other pets) directed and/or delivered by health, education and human service professionals. AAT focuses on enhancing physical, cognitive, behavioral and/or socio-emotional functioning of the particular human recipient and, to evaluate its efficacy, the intervention progress is measured by several physiological and psychological parameters included in professional documentation [17].

The delivery of AAT can be a valuable approach in the most varied contexts: from applications for children (autism and hyperactivity) and adolescents (anorexia and self-esteem problems) to intervention with patients with neuromotor and sensory disabilities and to relieve the loneliness and depression that may arise in senile age [18,19,20,21].

In Italy, AAT has been recognized as an official care by the Prime Minister Decree of 28 February 2003. In the case of elderly institutionalized individuals, a lack of social stimuli can aggravate isolation due to senile age and the illness, leading to psychological, emotional and cognitive degeneration [22]. In such a condition, the presence of an animal could fulfil the useful task of a social catalyst. Some international research has verified the possibility of using AAT as a supportive therapy in subjects with Alzheimer’s disease. Data collected in these studies, though heterogeneous and still in need of further confirmation, indicate that AD patients respond positively to AAT, with a general transient improvement in their psychophysical condition [23]. In addition, caregivers would also benefit indirectly from the interaction between patients and animals. In 2001, Kanamori and colleagues’ study [24], based on a comparison of a group of patients with dementia (seven subjects, five affected by AD and two by vascular dementia) participating in AAT and a control group (twenty subjects, of which seven were affected by AD and thirteen by vascular dementia), showed encouraging results. In fact, the group of treated subjects had a slowdown in cognitive decline, an increase in functional daily life skills (compared to controls that showed a deterioration) and a reduction in behavioral and stress disorders. In the following years, other studies conducted on small groups of patients confirmed these findings, including, among other things, a reduction in pathological behaviors (particularly in daytime hours), reduced agitation and an increase in some socializing behaviors (smiles, interactions and verbalization) [25,26]. However, the sporadicity of studies in this field and the presence of various methodological problems have, as a consequence, caused there to still be no certainty regarding the specific effects of AAT. To provide additional data on this topic, we investigated the effects of AAT on 24 residents (12 of which were affected by Alzheimer’s disease) of a rest house in northern Italy for three months. Considering that a faster cognitive decline and depression (mild to severe) are widely accepted as markers of the disease progression [27], neurocognitive and depressive states were investigated and measured by MMSE and GDS tests, respectively. Moreover, based on literature evidence reporting the so-called “head to heart” connection, i.e., a link between various cardiovascular abnormalities (heart failure, coronary artery disease, atrial fibrillation and vasculopathy) and Alzheimer’s disease [28,29], three physiological cardiac parameters (heart rate and systolic and diastolic blood pressure) were assessed to evaluate and monitor potential health benefits in residents receiving AAT.

## 2. Materials and Methods

### 2.1. Ethical Approval and Informed Consent

An informative meeting was organized with all residents of the rest house, their families and the staff members. The study was approved by the local Ethical Committee. After study protocol explanation, informed consent was signed by all participants or, in the case of cognitively impaired persons, by a proxy.

### 2.2. Study Population

The study involved 24 (18 women and 6 men) older people (average age = 83 ± 6.10 years), residing in a rest house in a wealthy area of northern Italy. Of them, 12 residents were not affected by AD (nAD) and 12 were diagnosed as affected by AD (AD). The nAD and AD groups were dichotomized in 2 groups: CONTROL group (Ctrl), which included residents who did not receive AAT, and the AAT group (AAT), which included residents receiving AAT. Hence, the resulting four groups were as follows: AD-AAT (*n* = 6), AD-Ctrl (*n* = 6), nAD-AAT (*n* = 6) and nAD-Ctrl (*n* = 6). The study protocol was agreed with several professionals of the rest house (the director as project coordinator, a coordinator, two doctors, a veterinarian, two physiotherapists, six nurses, a psychologist, two educators, a speech therapist and a social worker) and five trained and licensed dog operators with the task of drafting, defining and carrying out AAT sessions. Residents, selected by diagnosis (previously performed and specified in their clinical records), maintained their daily life style and rhythms throughout the duration of the study. Inclusion criteria were: homogeneity for the presence/absence of probable diagnosis of Alzheimer’s disease. Diagnoses were based on the International Statistical Classification of Diseases and Related Health Problems, 10th Revision (ICD-10) criteria [30]. Exclusion criteria from the study were: (i) allergies to dogs; (ii) behavioral disorders (aggressive behavior); (iii) condition of immunodepression.

### 2.3. Experimental Design and Data Collection

The project lasted 82 days (from 3 February to 24 April 2019, prior to COVID-19 pandemic), during which twelve residents (nAD-AAT and AD-AAT groups) of the rest house received AAT. Twelve residents (nAD-Ctrl and AD-Ctrl groups) were controls. AAT intervention consisted of one 45-minute AAT session per week over ten weeks. Altogether, nAD-AAT and AD-AAT residents interacted with five dogs (two Cocker Spaniels, two Labrador Retrievers and one mongrel) on rotation. Only one dog (with its own operator) was present in each session. AD-Ctrl and nAD-Ctrl residents were not allowed to see the animals nor to interact with them. The dogs were temperament tested and checked by veterinarians to ensure their health and current vaccination status. Dogs were professionally trained to execute basic commands and were accustomed to joining the AAT session. They were also trained to be unaffected by sudden noises, being gently and roughly caressed and being brushed. Dogs were already familiar with people affected by somatic and mental diseases, and with subjects using locomotion equipment. All criteria suggested by the Italian declaration of values and principles of pet relationships (‘Carta Modena 2002′) were fulfilled (https://www.salute.gov.it/imgs/C_17_pagineAree_356_listaFile_itemName_0_file.pdf accessed on 15 January 2019).

Dog operators had adequate knowledge about the behavior, needs, health and indicators and regulation of stress of the animals involved. Each dog had its own operator who was also trainer and responsible for the dog.

Sessions (T1–T10) took place in a spacious (about 80 sqm), quiet, bright and heated room, usually dedicated to sports and physiotherapy, with residents arranged to form a circle. The main goal of AAT was to provide an experience that could promote interpersonal relationships and communication, thereby increasing socialization levels and stimulating both residual cognitive–behavioral abilities and emotional–affective skills. In accordance with this, AAT sessions were set up to obtain a gradual involvement of residents through three different steps: (1) no physical contact with the animal (residents were only allowed to observe the operator–dog interaction); (2) indirect resident–dog interaction (dog on a lead held by the operator); (3) direct resident–dog interaction, under the watchful eye of the operator. Activities included close contact with the dog, talking, cuddling and playing (such as throwing balls).

In order to evaluate the physiological and clinical effect of AAT on AD residents, systolic and diastolic blood pressure (SP and DP, respectively) and heart rate (HR) were measured before each session (pre) and after the end of each session (post) once a week over ten weeks, and 15 days after the end of session 10, as follow-up (T+15).

### 2.4. Assessment of Neuropsychological Function

The evaluation of neurocognitive and depressive states (neuropsychological parameters) was performed using the Mini Mental State Examination (MMSE) and the Geriatric Depression Scale (GDS), respectively [31,32], administered by the psychologist of the rest house. MMSE is an 11-item tool widely used for neurocognitive research and is particularly focused on assessing spatial–temporal orientation, memory, attention, calculus and language skills; the lowest the total score, the higher the degree of cognitive deterioration (24–30: no cognitive impairment; 20–23: mild cognitive impairment; 10–19: moderate cognitive impairment; ≤9: severe cognitive impairment). The 30-item GDS is an external and self-administered tool for monitoring the degree of depression in older people; the higher the score, the more severe the depression (0–9: normal; 10–19: mild depression; 20–30: severe depression). The dog operators, the psychologist and the staff responsible for the rest house were blind to the results obtained in the assessment. The psychologist administering MMSE and GDS was blind to assignments to the case or the control group.

MMSE and GDS were administered before session 1 (T1), after the end of session 5 (T5), after the end of session 10 (T10), and 15 days after the end of session 10 (T+15). Additionally, as qualitative information, the AAT effects on behavioral parameters were evaluated by a questionnaire checklist prepared by the psychologist (Appendix A) and fulfilled by the operators at the end of session 10, in order to monitor the emotional and affective states, along with the motor, communicative, attention and executional functions showed by enrolled subjects.

### 2.5. Statistical Analysis

In order to analyze the effect of therapy for each considered variable (SP, DP, HR, MMSE and GDS), we applied a 4-way ANOVA model (including 2 ways for repeated measures): diagnosis (AD vs. nAD), therapy (AAT vs. Ctrl), sessions (T1–T10) and pre–post (parameter values measured before and after each session). To verify the persistence of the effects after 15 days from the end of the therapy period, we applied a similar 4-way ANOVA model (including 2 ways for repeated measures): diagnosis (AD vs. nAD), therapy (AAT vs. Ctrl), sessions (T10 and T+15) and pre–post. Information for each main effect and interaction possible in the design was given, with a *p* value < 0.05 indicating statistical significance. In addition, for all considered variables, a 3-way multivariate (general linear model) analysis, including diagnosis (AD vs. nAD), therapy (AAT vs. Ctrl) and session (T1), was performed considering parameter values measured before the beginning of the therapy cycle, with *p* < 0.05 indicating statistical significance. Moreover, in order to compare the four groups at initial and final points of the therapy cycle, a one-way ANOVA analysis was performed (with post hoc Bonferroni test) considering each parameter at T1 and T10. All the analyses were performed using SPSS (release 25.0, IBM, Armonk, NY, USA).

## 3. Results and Discussion

All enrolled residents were affected by cardiac dysfunction, vasculopathies, hypertension and an abnormal respiratory function. However, they showed normal cardiac parameters (systolic and diastolic blood pressure = 125 ± 18 mmHg and 71 ± 11 mmHg, respectively; heart rate = 75 ± 19 beats/min). Before the beginning of the therapy cycle, the mean values of all parameters measured at T1 for all participants of all groups were compared. Within each diagnosis group, participants were quite homogenous for the clinical parameters under investigation, with the exception of AD residents for the DP value (AD-AAT vs. AD-Ctrl, 83.33 ± 14.14 vs. 63.33 ± 5.50, *p* = 0.008). No differences were found between nAD and AD residents, except for the SP value (117.50 ± 10.78 vs. 133.33 ± 21.66, *p* = 0.03), whereas, as expected, cognitive deterioration and mild depression were found in AD residents in respect to nAD (MMSE score, 11.72 ± 4.27 vs. 22.45 ± 3.56, *p* < 0.0005; GDS score, 12.17 ± 3.64 vs. 9.83 ± 3.38, *p* = 0.049). Values (reported as mean ± standard deviation) of clinical and neuropsychological parameters measured for each group at the beginning of session 1 (T1) and after the end of session 10 (T10), also including a midterm evaluation at session 5 (T5) for MMSE and GDS scores, are reported in Table 1, along with values assessed (as a follow-up) 15 days after the end of the therapy cycle (T+15).

In order to interpret the effect of the therapy in AD residents, the interaction effects of the session by diagnosis by therapy were considered for each variable. As additional information, we also evaluated the AAT effect within each single session (interaction effect of pre–post by diagnosis by therapy).

**Figure 1:**
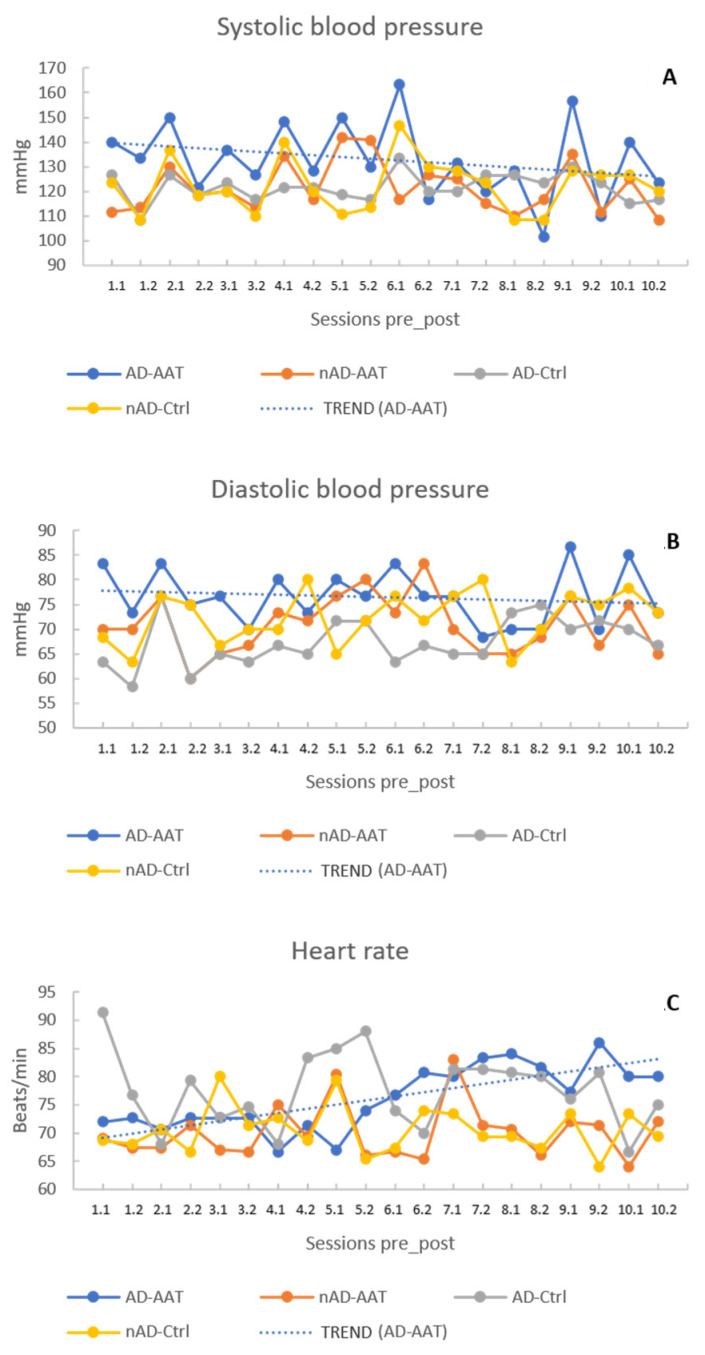
Kinetic of cardiac parameters. Systolic blood pressure (**A**), diastolic blood pressure (**B**) and heart rate (**C**) were monitored once a week, before (pre) and after (post) each session over 10 weeks. X axis: labels (1–10) correspond to sessions, pre and post are indicated as “.1” and “.2”, respectively.

In regard to AAT effects on systolic pressure, the analysis showed that, over ten weeks, the interaction of session by diagnosis by therapy accounted for 96% of SP variance (interaction effect: session * diagnosis * therapy, *p* < 0.0005; partial eta squared: 0.962). Both AD-AAT and nAD-AAT groups showed a decrease in SP values (AD-AAT: from 140.00 ± 23.81 to 123.33 ± 19.02; nAD-AAT: from 111.67 ± 3.93 to 108.33 ± 11.91), but with a different trend. Moreover, within the AD-AAT group, when comparing SP values before and after each session, it was evident that at the end of each session, the SP value was considerably lower than that measured before the beginning of the session (Figure 1A). This suggests a transient significant beneficial effect of AAT in reducing SP values within each single session in addition to the overall positive effect in pressure reduction over the entire 10-week therapy cycle (interaction effect: pre–post * diagnosis * therapy, *p* < 0.0005; partial eta squared: 0.473). Moreover, considering that in the AD-AAT and nAD-AAT groups SP values were significantly different at T1 (140.00 ± 23.81 vs. 111.67 ± 3.93, *p* = 0.046) but no longer at T10 (123.22 ± 19.02 vs. 108.33 ± 11.91, *p* > 0.05), in our opinion this finding could suggest that AAT, although effective in both groups, seemed to exert a greater impact on systolic blood pressure reduction in AD residents.

In the case of diastolic blood pressure, the interaction of session by diagnosis by therapy accounted for almost 93% of the DP variance (interaction effect: session * diagnosis * therapy, *p* < 0.0005; partial eta squared: 0.926). In both groups receiving the therapy, DP values decreased, although with a different trend (AD-AAT: from 83.33 ± 14.14 to 73.33 ± 14.02; nAD-AAT from 70.00 ± 9.68 to 65.00 ± 8.46). Taking into account that DP mean values were in line with normal cardiac values (see Table 1), the fact that the effect of the therapy on diastolic pressure, although significant, was lower than that on systolic pressure was not surprising (interaction effect: session * therapy, *p* = 0.047 vs. 0.003). Diastolic pressure values measured before and after each session are plotted in Figure 1B.

Concerning the heart rate values, the interaction of session by diagnosis by therapy accounted for 91% of the HR variance (interaction effect: session * diagnosis * therapy, *p* < 0.0005; partial eta squared: 0.912). AD-AAT and nAD-AAT residents showed an increase in the heart rate—although still within the normal range—but with a different trend (AD-AAT from 72.00 ± 9.96 to 80.00 ± 20.83; nAD-AAT from 69.00 ± 13.49 to 72.00 ± 8.32). Actually, whether this physiological response is indicative of positive excitement or of potentially negative stress remains an open question. However, the observed positive effect (i.e., reduction) on systolic and diastolic blood pressure would seem to corroborate the hypothesis that human–animal interactions could contribute to improve the functioning of the cardiovascular system [33,34,35,36,37].

**Figure 2:**
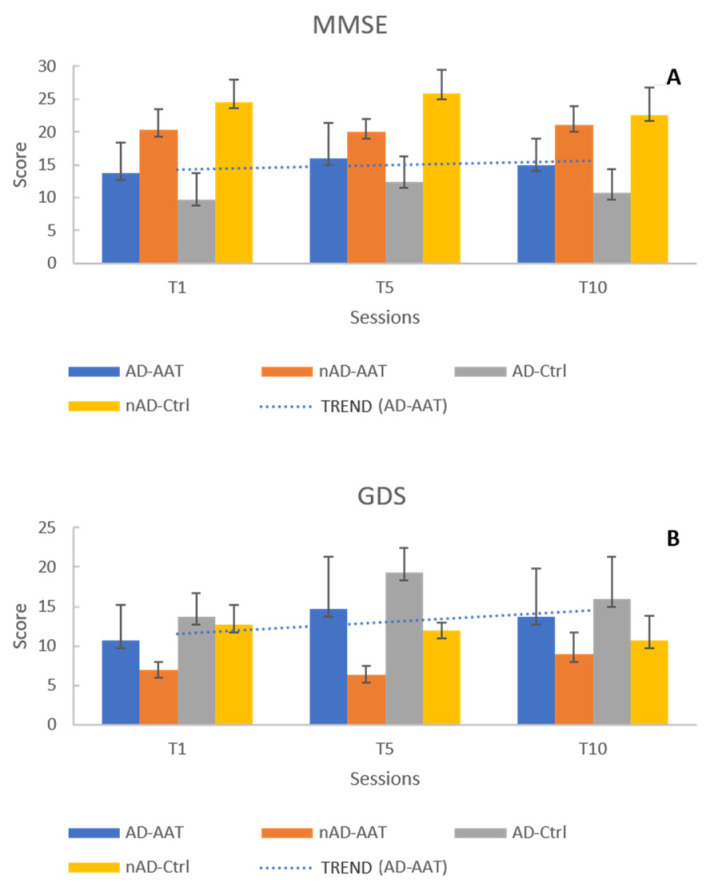
Evaluation of neurocognitive and depressive state performed by the Mini Mental State Examination (MMSE) and the Geriatric Depression Scale (GDS), respectively. MMSE (**A**) and GDS (**B**) scores were assessed before the beginning of session one (T1), after the end of session 5 (T5) and after the end of session 10 (T10).

For MMSE scores, the interaction of session by diagnosis by therapy was not significant, but the interaction of session by therapy accounted for 28% of the MMSE variance (interaction effect: session * therapy, *p* = 0.044; partial eta squared: 0.281). Both AD-AAT and nAD-AAT residents showed a similar mild improvement (Figure 2A) of the neurocognitive impairment. Since we did not find a significant interaction effect regarding session * group * therapy (*p* > 0.05), we believe that, at least in this case, AAT did not have a specific effect on AD residents receiving the therapy. Nevertheless, our results were encouraging concerning the use of animal-assisted therapy in the treatment of mild cognitive deficits deriving from senile age.

Considering the depression level, evaluated by the GDS scores (Figure 2B), no significant effects could be observed, at least over the 10 weeks of treatment.

Observations at follow-up showed that, for the systolic and diastolic blood pressure and neurocognitive state, the beneficial effects of AAT persisted (at least) up to 15 days after the end of session 10 (interaction effect: session * diagnosis * therapy, *p* > 0.05).

In addition, as qualitative information, data from questionnaire checklist (Appendix A) suggested that, in AD residents, AAT also had beneficial effects on stress reduction and produced positive emotional–affective, motor–executive, socio-communicative and cognitive–attentive behavioral changes. In fact, interaction with the animal was certainly a positive stimulus as, from the first meetings, residents were able to overcome fear and anxiety, showing pleasant sensations such as calm, self-esteem, empathy and affection. Overall, the present results added new insights to the potential of AAT to: (i) modulate the systolic and diastolic blood pressure and heart rate; (ii) reduce stress levels; (iii) improve cognitive functioning and enhance psychological well-being. That could be the reason why one of the areas in which AAT finds more space and effectiveness is that of care facilities for geriatric patients and patients with different levels of dementia, where it can be used to control behavioral disorders and address health problems that may gradually arise [38,39]. Results offered by AAT have attracted the attention of many researchers in the field of Alzheimer’s disease, suggesting that, in patients with dementia, interaction with animals could have a positive influence, as it helps to keep busy, stimulates physical activity and supports emotional balance. Since studies carried out so far are extremely heterogeneous and show controversial results [40], it is not possible to draw any definitive conclusions on AAT effects on Alzheimer’s disease. However, taking into account that, at the moment, there is no cure for dementia, the main purpose of AAT should be addressed to reduce its burden to both patients and caregivers. On that side, the presence of a dog may be helpful to reduce anxiety and stimulate residual abilities.

## 4. Conclusions

In conclusion, concerning the systolic and diastolic blood pressure modulation and neurocognitive impairment, our results seemed to confirm a general beneficial effect of AAT, which could also be observed in AD residents, whereas no improvement of the depressive state was detected. The interpretation of AAT’s effect on the heart rate still remains elusive. However, to better investigate animal-assisted therapy effects on Alzheimer’s disease, besides the clinical and neuropsychological markers examined in the present study, additional parameters should be evaluated. It would be of great interest to monitor neuroendocrine parameters such as beta-endorphins (to evaluate the state of well-being), blood or salivary cortisol levels (stress indicators) and the plasma concentration of some proinflammatory cytokines (TNF-alpha, IL-6, IL-1-beta and IFN-gamma), which are particularly relevant in Alzheimer’s disease, whose assessment could provide useful indications on AAT efficacy and, above all, a preliminary insight on its action mechanisms. Therefore, studies involving a higher number of subjects, for a significant period of time (at least one year) and monitoring suitable clinical parameters are certainly needed and warmly welcomed.

## Figures and Tables

**Table 1:** Means ± standard deviation (SD) for the demographic, clinical and neuropsychological parameters: systolic pressure (SP), diastolic pressure (DP), heart rate (HR), Mini Mental State Examination (MMSE) and Geriatric Depression Scale (GDS) in non-Alzheimer’s disease (nAD) and Alzheimer’s disease (AD) residents receiving animal-assisted therapy (AAT) or not receiving animal-assisted therapy (Ctrl). T1: before session 1; T5: after session 5; T10: after session 10; T+15: 15 days after the end of the therapy cycle.

Demographic, Clinical and Neuropsychological Parameters	nAD			AD		
	Ctrl	AAT	Total	Ctrl	AAT	Total
Age (years)	79.33 ± 3.78	84.67 ± 8.38	82.00 ± 6.80	86.38 ± 3.88	81.67 ± 6.06	84.00 ± 5.43
Gender (F/M)	4/2	4/2	8/4	4/2	6/0	10/2
SP (mmHg)						
T1	123.33 ± 12.58	111.67 ± 3.93	117.50 ± 10.78	126.67 ± 18.93	140.00 ± 23.81	133.33 ± 21.66
T10	120.00 ± 9.57	108.33 ± 11.91	114.17 ± 11.97	116.67 ± 14.43	123.33 ± 19.02	120.00 ± 16.47
T+15	140.00 ± 35.85	146.67 ± 21.45	143.33 ± 28.38	135.00 ± 42.90	135.00 ± 34.08	135.00 ± 36.94
DP (mmHg)						
T1	68.33 ± 4.27	70.00 ± 9.68	69.17 ± 7.18	63.33 ± 5.50	83.33 ± 14.14	73.33 ± 14.62
T10	73.33 ± 5.65	65.00 ± 8.46	69.17 ± 8.12	66.67 ± 11.04	73.33 ± 14.02	70.00 ± 12.53
T+15	85.00 ± 8.97	83.33 ± 3.14	84.17 ± 6.46	76.67 ± 11.76	75.00 ± 5.62	75.83 ± 8.83
HR (beats/min)						
T1	68.67 ± 6.77	69.00 ± 13.49	68.83 ± 10.18	91.33 ± 29.25	72.00 ± 9.96	81.67 ± 23.15
T10	69.33 ± 4.55	72.00 ± 8.32	70.67 ± 6.54	75.00 ± 16.43	80.00 ± 20.83	77.50 ± 18.08
T+15	68.33 ± 5.65	76.00 ± 8.00	72.17 ± 7.72	84.33 ± 31.49	80.33 ± 26.27	82.33 ± 27.73
MMSE (score)						
T1	24.57 ± 3.09	20.33 ± 2.75	22.45 ± 3.56	9.77 ± 3.58	13.67 ± 4.26	11.72 ± 4.27
T5	25.90 ± 3.20	20.00 ± 1.73	22.95 ± 3.94	12.43 ± 3.42	16.00 ± 4.84	14.22 ± 4.41
T10	22.60 ± 3.79	21.00 ± 2.67	21.80 ± 3.23	10.77 ± 3.21	15.00 ± 3.59	12.88 ± 3.93
T+15	26.50 ± 3.01	22.33 ± 4.29	24.42 ± 4.15	13.10 ± 5.07	17.00 ± 2.58	15.05 ± 4.34
GDS (score)						
T1	12.67 ± 2.25	7.00 ± 0.89	9.83 ± 3.38	13.67 ± 2.73	10.67 ± 4.03	12.17 ± 3.64
T5	12.00 ± 0.89	6.33 ± 1.03	9.17 ± 3.10	19.33 ± 2.73	14.67 ± 5.96	17.00 ± 5.05
T10	10.67 ± 2.88	9.00 ± 2.37	9.83 ± 2.66	16.00 ± 4.73	13.67 ± 5.47	14.83 ± 5.02
T+15	9.67 ± 3.72	7.33 ± 1.37	8.50 ± 2.94	14.33 ± 3.62	12.33 ± 3.39	13.33 ± 3.50

## Appendix A. Questionnaire Checklist

Date:
Place:

User name and surname:
Center of origin:
Filling operator name:
Dog operator name:
Dog name:

Emotional State

Indicate the emotional reactions manifested in contact with the dog:

- Fear
- Discomfort
- Indifference
- Pleasure
- Relaxation
- Increased ability to concentrate
- Other (specify: )

Motor Level

During the activity the subject:

- Moves in a rigid way
- Moves in a stereotyped way
- Opposes resistance
- Is relaxed
- Follows the dog in its movements
- Looks for contact with the dog
- Demonstrates greater fluency
- Stiffens
- Is involved
- Is detached
- Moves away

Affective Appearance (Relational aspects with the dog)

The subject:

- Recognizes the dog
- Looks for contact with the dog
- Looks for the dog with his eyes
- Is indifferent
- Prefers an activity to do with the dog (lead it, play with it, do exercises ...)
- Prefers emotional contact (pamper him, brush him, feed him ...)
- Refuses contact with the dog
- Touches the dog on request
- Does not touch the dog even on request

Emotional state prior to the session: ____________________________________________
Emotional state immediately after the session: _________________________________________
Emotional state during the rest of the day: _________________________________________
Communication Level

Main type of communication:

- Verbal communication
- Non-verbal communication

The session is interrupted because:

- The dog operator interrupts
- The user loses interest or leaves
- Time is over

How long does the subject stay focused?

- The whole session
- Only during certain activities (specify: )
- A little

Between sessions, the subject:

- Talks about the dog
- When talking about the dog, he/she expresses joy and a desire to see it again
- When talking about the dog, he/she is indifferent
- Expresses fear and anxiety at the thought of seeing the dog again

Is the subject able to carry out the assigned deliveries?

- Yes
- No

Comments:
